# Identification and Determination of Dimensions of Health-Related Quality of Life for Cancer Patients in Routine Care – A Qualitative Study

**DOI:** 10.3389/fpsyg.2022.824099

**Published:** 2022-03-09

**Authors:** Theresa Schrage, Mirja Görlach, Holger Schulz, Christiane Bleich

**Affiliations:** Department of Medical Psychology, University Medical Center Hamburg-Eppendorf, Hamburg, Germany

**Keywords:** cancer, health-related quality of life, quality of life measurement, focus groups, psycho-oncology, qualitative content analysis

## Abstract

**Purpose:**

Continuous patient-reported outcomes (PROs) to identify and address patients’ needs represent an important addition to current routine care. The aim of this study was to identify and determine important dimensions of health-related quality of life (HrQoL) in routine oncological care.

**Methods:**

In a cross-sectional qualitative study, interviews and focus groups were carried out and recorded. The interviewees were asked for their evaluation on HrQoL in general and specifically regarding cancer treatment. The material was transcribed and analyzed using qualitative content analysis based on Mayring. The results were reviewed in an expert discussion.

**Results:**

Interviews with patients (*N* = 28) and clinicians (*N* = 4), as well as five focus groups with clinicians (*N* = 18) were conducted. Initially, nine deductive and two inductive categories on HrQoL were built. Four categories (*partnership/sexuality*, *spirituality/religiousness*, *health perception*, and *overall health*) were excluded following the qualitative content analysis because they were hardly or not at all mentioned by participants. Following on from the analysis of the expert discussion, one dimension was added (*dignity*), and two further categories were excluded (*mobility* and *feeling of security in treatment*). The resulting system consisted of six dimensions: *emotional health, physical ailments, autonomy, social functionality, dignity*, and *resources.*

**Conclusion:**

The identified dimensions of HrQoL in routine oncological care were found to differ from those used in existing HrQoL measurements for (cancer) patients. Further research is needed to test and evaluate the presented structure in a larger sample of cancer patients to further assess its psychometric properties.

## Introduction

During treatment many cancer patients experience impairments in health-related quality of life (HrQoL) (Mehnert et al., 2014). However, these impairments are often not sufficiently identified and addressed in clinical routine (Snyder et al., 2012; Antunes et al., 2013). In the context of improving oncological patient-centered care, continuous patient-reported outcomes (PROs) represent an important addition to current routine care. PROs are critical in order to identify and address the patient’s needs (Snyder et al., 2012; Basch et al., 2016).

Cancer patients can experience many forms of psychological strain during their illness and treatment. Their relationships and their social life a.o. can be affected (Guida et al., 2019; Roick et al., 2019). As a consequence, psychosocial distress and psychological comorbidities (i.e., anxiety and depressive disorders) can occur or be amplified during or after treatment (Singer et al., 2010). Moreover, cancer patients often experience severe side effects, such as pain, fatigue, weak immune system, indigestion, or sexual dysfunction as a result of their treatment (Aigner and Stephens, 2016). Taking into consideration the 10 year survival rate of 57–61% (Barnes and Kraywinkel, 2016), it is clear that many cancer patients also experience suffering and considerable functional limitations on a daily basis over a long period of time with (Barnes and Kraywinkel, 2016). Correspondingly, the negative impact on the patients’ HrQoL can be immense. This emphasizes the importance of focusing on HrQoL in clinical practice and research. In the context of patient-centeredness (Scholl et al., 2014), monitoring a patient’s HrQoL can also create an opportunity to actively influence the clinician-patient relationship. In order to measure the patients HrQoL it is important to know what HrQoL means to health care providers and patients and how to assess it.

Several dimensional systems and questionnaires on HrQoL exist which can be related to these questions. In the last 30 years of HrQoL research the dimensions in Table 1 a. o. have been identified to be important in defining HrQoL.

**TABLE 1:** Dimensions of HrQoL measurements.

*Dimensions*	*Questionnaires*
*Functionality*	EORTC QLQ-C30 (Aaronson et al., 1993), FACT-G (Cella et al., 1993), SF-36 (Ware and Sherbourne, 1992), Distress-Thermometer (DT) (Mehnert et al., 2006)
*Pain/physical ailments*	EORTC QLQ-C30 (Aaronson et al., 1993), SF-36 (Ware and Sherbourne, 1992), Nottingham Health Profile (NHP) (Kohlmann et al., 1997), EQ-5D (Herdman et al., 2011), PROMIS (Cella et al., 2010), WHOQOL-100 (Gunzelmann et al., 2002)
*Autonomy*	EQ-5D (Herdman et al., 2011), WHOQOL-100 (Gunzelmann et al., 2002)
*Mobility*	NHP (Kohlmann et al., 1997), EQ-5D (Herdman et al., 2011), WHOQOL-100 (Gunzelmann et al., 2002)
*Emotional health*	WHOQOL-100 (Gunzelmann et al., 2002), PROMIS (Cella et al., 2010), DT (Mehnert et al., 2006), FACT-G (Cella et al., 1993), EQ-5D (Herdman et al., 2011), EORTC QLQ-C30 (Aaronson et al., 1993)
*Health perception*	PROMIS (Cella et al., 2010), SF-36 (Ware and Sherbourne, 1992)
*Spirituality/religiousness*	WHOQOL-100 (Gunzelmann et al., 2002), DT (Mehnert et al., 2006)
*Partnership/sexuality*	WHOQOL-100 (Gunzelmann et al., 2002), DT (Mehnert et al., 2006)
*Overall health*	SQLI (Spitzer et al., 1981)

Table 1 provides a brief overview of dimensions identified for measuring HrQoL. The European Organization for Research and Treatment of Cancer (EORTC) for example developed a core questionnaire [QLQ-C30 (Aaronson et al., 1993)] with a modular approach for all cancer patients (Aaronson et al., 1993). Another comprehensive measurement for cancer patients is the Functional Assessment of Cancer Therapy – General (FACT-G) (Cella et al., 1993) which consists of four main dimensions and 27 items of HrQoL (Bonomi et al., 1996). In addition to cancer specific questionnaires there are also generic health related instruments, such as the Short Form-36 Health Survey (SF-36) (Ware and Sherbourne, 1992) and WHOQOL-100 (Gunzelmann et al., 2002). What needs to be considered in this context is that oncological patients in general are older and often strongly affected by the intensive treatments, e.g., chemotherapy or stem cell transplantation. A comprehensive questionnaire like the EORTC QoL-C30 (Aaronson et al., 1993), however, often requires more capacity (e.g., time, scope) than available on both the patient’s and clinician’s side (Boyce et al., 2014), especially if the patient is to be questioned several times during treatment to monitor HrQoL progress. Furthermore, the complex assessment of questionnaires and unsightly number of items as well as difficulties in evaluation by health care providers are hindering the effective use of HrQoL measurements in clinical routine (Bascioni et al., 2005; Boyce et al., 2014).

Shorter HrQoL instruments, however, are often generic [EQ-5D (Herdman et al., 2011), Distress Thermometer (DT) (Mehnert et al., 2006)] and miss cancer specific HrQoL dimensions (Robbeson et al., 2018). They also often do not fit in a hospital setting since they were created for the outpatient setting, research setting or other purposes. For a frequently used measurement in routine care it is important to address the patients’ needs in an effective manner to provide the best possible care. What HrQoL means for in- and outpatients in routine cancer care today is an essential research question which until now has not been fully answered.

To answer this research question, this study was undertaken to develop a short health-related quality of life measurement for the recurrent use (monitoring) in routine cancer care. Within the framework of patient-centeredness this new measurement aims to support the clinician-patient relationship. Literature research, interviews, focus groups and an expert discussion were conducted. On these grounds, a dimensional system on HrQoL in cancer patients in routine care was developed. The items as well as the psychometric analysis (Schrage et al., in prep) and the implementation in clinical routine (Görlach et al., in prep) of the developed questionnaire will be outlined in subsequent publications.

## Materials and Methods

This study was part of the project “PRO-ONKO-Routine” (Schrage et al., 2020) and presents the results from the first qualitative phase of the project. The project was registered at OSF – Open Science Framework (Open Science Framework [OSF], 2019).

### Study Design

This was a cross-sectional qualitative study including interviews, focus groups and an expert discussion with oncological patients diagnosed with different cancer entities and oncological clinicians in a German university medical center. The “Standards for Reporting Qualitative Research (SRQR)” (O’Brien et al., 2014) was used as reference. It was approved by a local research ethics committee, (no. PV5636). All participants provided written consent. This study began in December 2017 and ended in April 2018.

To develop relevant dimensions of HrQoL for cancer patients, this study was conducted in five steps. The first step included interviews with both adult in- and out-patients with heterogenic cancer diagnoses and oncological health professionals. We decided to conduct interviews with patients to be mindful of the in-patient’s wellbeing while still being able to explore the research topic in depth. 28 interviews were planned to reach theoretical data saturation.

In the second step of the study five focus groups on relevant dimensions of HrQoL were conducted with oncologists, oncological nurses and psycho-oncologists, to facilitate further discussion and exchange. In the interviews and focus groups patients and clinicians were asked for their evaluation on HrQoL in general and specifically regarding cancer treatment.

A qualitative content analysis based on the transcripts of the interviews and focus groups was performed as a third step.

In the fourth step of the study, the outcome of the qualitative data analysis was presented to a group of eight experts from the Hamburg metropolitan area. The goal was to hear the perspectives on the now reduced but still large amount of information on HrQoL from experts in various related fields. The aim of this expert discussion was to place the information and outcomes in the context of the comprehensive oncological health care. Experts constituted psycho-oncologists, oncologists, a patient representative (leader of a self-help group), quality of life scientists, staff nurses, a representative of the quality management and a representative of a statuary health insurance company. They were presented with the preliminary results of the qualitative content analysis (step 3), interviews and focus groups. Every category was introduced and debated in an open discussion. In contrast to the focus groups (step 2), no new material on HrQoL was generated. Instead, the experts’ assessment of the categories (step 3) and the use of these as dimensions in a questionnaire were sought after. Finally, a consensus-based statement was formed for every presented category.

Based on the outcomes of step 4 and considering the current state of research, the dimensional system measuring HrQoL was developed in a fifth step. Psychometric testing of the questionnaire was conducted in a subsequent study.

### Cooperation Partners

Cooperation partners in this study were the II. Medical Clinic and Polyclinic, the Department of Gynecology, the Department of Radiotherapy and Radiation Oncology, the Department of Stem Cell Transplantation and the Department of Medical Psychology of the University Medical Center Hamburg-Eppendorf. All five departments are members of the University Cancer Center Hamburg (UCCH).

### Recruitment of Participants

Patients were recruited and equally stratified from the five cooperating departments. Patients fit for interviewing (not sedated and able to speak) were chosen by medical staff. The appointed patients were asked to participate and interviewed by MG and TS (step 1). Selection criteria were a cancer diagnosis, sufficient language skills in German and no severe cognitive or verbal impairments interfering with their ability to give informed consent and participate in the interview. Participants for the focus groups (step 2) also originated from the five cooperating departments. The focus groups were stratified by profession (nursing and medical profession) and, for physicians, by professional position (residents and attendings). For the expert discussion (step 4), experts or expert groups from different fields of expertise were addressed and invited to partake.

### Measurements

Every patient was asked to fill out a sociodemographic questionnaire about gender, age, family status, education and living situation. The sociodemographic questionnaire for clinicians contained questions about age, oncological specialty and years of experience in oncological care.

For interviewing patients in study step 1, a semi structured interview guide (Supplementary Appendix A) was developed based on Helfferich (2009) which consisted of one main question and subquestions concerning relevant dimensions of HrQoL to be assessed in routine care. The main question asked was: “**If you imagine that your current doctor asks you about your quality of life, physical and mental distress, what would be important for you in this matter?/What should not be omitted?**”. With this main question the patients were asked on the one hand about aspects of HrQoL relevant to them and on the other hand about a ranking of aspects of HrQoL which were most important to the patients. For structuring focus groups in step 2, a guideline of eight questions (Supplementary Appendix B) was issued referring to Barbour (2014) which included the same main question as the interview guide. The expert discussion (step 4) was moderated by one of the authors (HS) (Li et al., 2018). HS is a clinical psychologist with experience in moderating discussions. No stimulus material was provided for the experts beforehand.

### Qualitative Analysis

Interviews (step 1), focus groups (step 2) and expert discussion (step 4) were carried out by scientists, recorded and afterward transcribed by study staff. The qualitative data of the interviews and focus groups was structured via MAXQDA 10^1^ and analyzed using a qualitative content analysis (step3) based on Mayring (2008). This qualitative method was selected because of its flexibility toward the material and at the same time theory-guided procedure. Deductive-inductive category application was used: deductive main-categories [generated through literature research (Table 1)] and inductive sub-categories (derived from text analysis), including the option of additional inductive main categories. The deductive main categories formed the basic framework of the analysis. They were derived from literature research on general and cancer specific HrQoL measurements. The dimensions of these standardized and validated measurements (Table 1) formed the deductive categories for our category system. By adding inductive categories, the study’s intent – identifying HrQoL dimensions of patients in cancer care in a hospital setting – could be pursued more thoroughly.

For the procedure of coding, one coding unit had the length of approximately one paragraph. MG and TS generated the category system by separately coding one transcript and discussing the results afterward. Two scientific student assistants then coded a transcript each with the resulting category system. Subsequently, the results were discussed with MG and TS. On these grounds, all other transcripts were analyzed using the same category system. Explicit descriptions and anchor samples were formulated for every category.

## Results

### Sample – Interviews

A total of 28 patients participated (female = 20, male = 8). Their mean age was 58 (*SD* = 16.6, range = 30–82). We interviewed 17 (60.7%) inpatients and 11 (39.3%) outpatients. Patients graduated after 10 years (*n* = 11, 39.3%) or after 12 and 13 years (*n* = 10, 35.7%) from secondary school and 7 (25%) patients graduated from university. Most patients (*n* = 19, 67.9%) were married or with permanent partnership. More than half of the participants (60.7%) lived in a large city and worked as full-time employees (53.6%). The average interview time was 20.2 min (*SD* = 12.4).

### Sample – Focus Groups

Eighteen clinicians (4 male, 14 female) of the age between 26 and 60 years with a mean age of 38 years (*SD* = 11.0) participated in five focus groups conducted between January and March 2018 in Hamburg (see Table 2). Their professional experience ranged from 2 to 30 years with a mean experience of 11 years (*SD* = 8.7). Nurses and residents took part in two groups each and psycho-oncologists took part in one focus group. Due to time constraints no senior physicians were able to take part in the focus groups. Instead, two senior physicians and two senior psycho-oncologists participated in interviews, which could be scheduled more flexible. The clinicians were employed at the five different cooperating oncological departments.

**TABLE 2:** Overview of conducted interviews und focus groups participants.

	Interviews	Focus groups
*Patients*	28	–
*Nurses*	–	8 (*n* = 2 groups)
*Residents*	–	5 (*n* = 2 groups)
*Attendings*	2	–
*Psycho-oncologists*	2	5 (*n* = 1 group)

### Sample – Expert Discussion

We conducted the expert discussion with eight participants in April 2018. The experts were psycho-oncologists, oncologists, a patient representative (leader of self-help group), quality of life scientists, staff nurses, a representative of the quality management of the UKE and a representative of a statuary health insurance company. The results of the expert discussion group were considered as expert consensus-based statements.

### Categories

Nine main categories from existing material (Spitzer et al., 1981; Ware and Sherbourne, 1992; Aaronson et al., 1993; Cella et al., 1993, 2010; Kohlmann et al., 1997; Gunzelmann et al., 2002; Mehnert et al., 2006; Herdman et al., 2011) were derived to depict HrQoL in cancer patients: *partnership/sexuality, spirituality/religiousness, health perception, emotional health, mobility, self-care/autonomy, pain/physical ailments, functionality (work, free time, social), overall health*.

Statements about the patients’ *partnership/sexuality* were not emphasized and rarely made:

“[…] to adjust more to my partner again […]” *(REC020, partnership/sexuality, 13:18:00).*

In terms of *health perception*, respondents were primarily concerned with the difference between the impact of the disease on themselves and the impact on their surroundings. This deductive category was also hardly applied during qualitative analysis.

“Yes, the possibility to think about other people again and to not always have the disease in mind. That is very important, I think. When you’re sick, you also think very egocentrically.” *(REC023, health perception, 14:43:00).*

The deductive categories *spirituality/religiousness* and *overall health* were not get mentioned at all.

On the contrary, the participants talked a lot about *emotional health* in oncological patients. Various aspects of *emotional health* were included, among of which anxiety and depressed mood were frequently mentioned:

“I just cried and worried, couldn’t sleep properly, things like that.” *(REC017, emotional health, 11:03:20).*

“It’s just totally frightening, this mammogram, that someone will tell me: it’s back again and you’ll have to be operated, again.” *(REC006, emotional health, 06:10:00*).

“When I was still on business trips, I had to take off my scarf at the security check and my wig slipped, then it fell off. It was, well, that was so awkward. I’m always afraid of what can happen when people see that my hair looks completely different, where I can’t decide who I tell and who doesn’t notice. That is already quite a burden.” *(REC013, emotional health, 13:16:57).*

The participants also talked about a sense of feeling lost and powerless.

“I sometimes had the feeling of helplessness because of that” *(REC003, emotional health, 11:21:00).*

“The burden is that I will have a different life from now on, that I will be limited, that I know it is not curable and that I always have that in my mind and always have to try that there is another me and that I still remain me, with this disease that will surely accompany me for the rest of my life.” *(REC023, emotional health, 14:42:00).*

Another part of emotional health focused on the perceived stigmatization of cancer and its effects on the patients:

“On the one hand, the stigmatization, that cancer is associated with death and dying, that you have the feeling that people change sides of the street, you hear that again and again.” *(REC001, emotional health, 20:57:00).*

Moreover, three other deductive categories could be rediscovered in the transcripts. One of them was *self-care/autonomy*. Autonomy and independence often were considered synonymous with freedom and quality of life:

“*Self-care/autonomy often goes hand in hand with mobility. Feeling of freedom is also important here, not being restricted in one*’*s actions*.” *(REC006, autonomy, 04:16:00).*

“Thus, quality of life mainly is food for patients with gastric carcinoma or esophageal carcinoma, but that is. The possibility of being able to feed oneself independently.” *(REC037, self-care/autonomy, 13:10:08).*

The second deductive category identified was pain and physical impairment. Pain, as well as physical suffering, was more frequently addressed by outpatients.

“Since I had one breast amputated, I have had pain for years. It’s a little better now, but it’s not gone. It’s quite restricting.” *(REC006, pain/physical ailments, 15:03:00).*

“I was not prepared for the fact that my intestines were permanently damaged by radiation and that I have had diarrhea for three years because of it.” *(REC003, pain/physical ailments, 08:19:00).*

The last deductive category of our analysis addressed the wide range of functionality. For a better overview we have created subcategories, work-related functionality, free time-related functionality and social functionality.

“Communication skills and interactive get-togethers, socializing, that actually helps.” *(REC026, functionality/social, 09:35:00).*

“*Family plays a special role in terms of quality of life.*” REC037, *social functionality*, 05:21:00.

**FIGURE 1:**
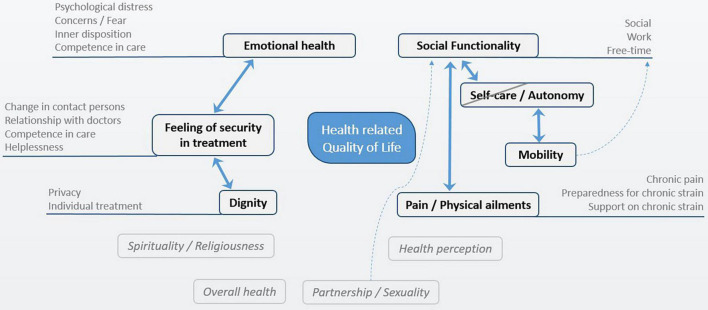
Qualitative content analysis: Categories of HrQoL in cancer patients.

Throughout the coding process, it became apparent that two further main categories were important to cancer patients and clinicians: *dignity* and *feeling of security in treatment*. Figure 1 provides an overview of the main categories and their subcategories used in this qualitative content analysis.

Cancer patients’ and clinicians’ views on HrQoL were consistent. Both parties named similar aspects to be important for themselves and for cancer treatment. They emphasized especially two aspects which were not already part of the deductive categories generated from the literature research. On the one hand, dignity was of great importance for HrQoL in cancer patients:

“*Because every patient is a human and not a number or a procedure.*” (*REC003, Dignity, 11:33:00).*

“*Yes, well, that is, I think something that has to do with quality of life being treated with dignity. I don*’*t need to be cuddled but treated as a human and it*’*s important that it is accepted that I have a kind of privacy.*” *(REC001, Dignity, 19:00:00).*

On the other hand, a feeling of security in treatment was noticeable and was often mentioned. This category included information on whether patients generally felt well looked after in the clinic and their treatment:

“*I have to say, I feel I’m in good hands. Everything is taken care of here, whether it is the sage tea or that they ask every day what I need.*” *(REC019, Feeling of Security in Treatment, 12:15:13)*.

The analysis also showed that four categories had no significant relevance for HrQoL: *partnership/sexuality*, *spirituality/religiousness*, *health perception*, and *overall health* (see Figure 1). These categories were hardly mentioned and therefore excluded from the dimensional system.

### Expert Discussion

The categories were separately talked about in detail during the expert discussion which revealed the following consensus:

*Pain/physical ailments*: Collectin informations about pain and physical ailments are very important for patients and a central focus area in treatment by clinicians. This is already reflected in the clinical daily routine already. As part of the treatment concept of the Comprehensive Cancer Center at the UKE, patients are asked about their pain levels every day. Furthermore, there already is a focus in treatment on physical ailments.
*Autonomy*: Emotional distress as a result of a lack of perceived autonomy need to be addressed in a consultation with a clinician. Even in a hospital setting conditions can be created which cater to a patient’s need for autonomy.
*Functionality*: Physical functionality especially, but also social and work-related functionality, can be represented by the other dimensions in this category system. For example, physical functionality can be merged into the physical ailments category. *Social functionality* holds great importance for both hospitalized patients and outpatients.
*Mobility:* Mobility is of importance though rather less in a hospital setting, as the interviews with patients showed. In this setting, mobility is subordinate to autonomy.
*Emotional health*: This dimension is important throughout the duration of the disease. It is noteworthy that at different stages of the disease different emotional strains appear. In a hospital emotional health can be negatively affected after patients are made aware of their diagnosis. For outpatients the fear of disease recurrence can be enhanced by regular check-ups.
*Dignity*: Views on dignity can differ between patient and clinician. The feeling of dignity is very subjective so that associated distress needs to be addressed in a consultation with a clinician. A high level of informedness in a patient can positively influence the feeling of dignity (this also is an important aspect in the framework of shared decision-making and patient-centeredness).
*Feeling of security in treatment*: Having trust in clinicians can make a big difference for patients. In the end, this can improve patients’ treatment because patients who feel secure tend to be more open to revealing personal information to clinicians. A high correlation with the dimensions dignity and emotional health can be observed here.
*Coping/Resources*: During treatment it is important to address whether there is a need for further support systems. A patient might experience, e.g., los emotional health and at the same time feel sufficiently supported by his or her family. This category could be viewed as a summarizing assessment for the HrQoL measurement and help in providing recommendations for action to clinicians later on.

**FIGURE 2:**
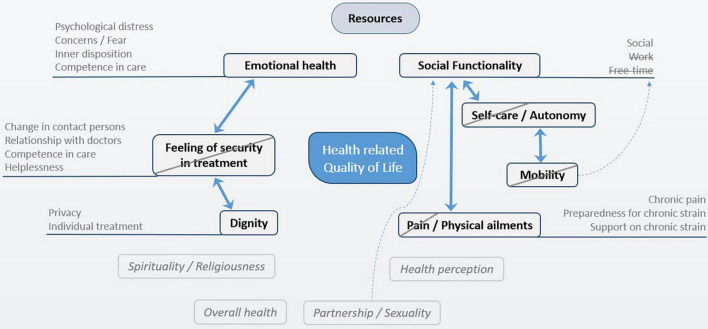
System of dimensions of HrQoL in cancer patients.

Following on from the expert discussion the dimensional system was further developed. Interrelations between categories (see continuous arrows Figure 2) were considered, especially to avoid redundancies which led to exclusion of two dimensions:

*Mobility*: This dimension is closely linked to autonomy and it can be concluded that as a stand-alone dimension it does not lead to a collection of information which provides added value in a hospital setting.
*Feeling of security in treatment*: This inductive category is of importance to patients and clinicians. In the face of high correlations and an overlap with the important dimensions of *emotional health* and *dignity*, it was, however, excluded.

Lastly, the dimension *pain/physical ailments* was restricted to *physical ailments* because pain is already assessed by clinicians on a daily basis. The *functionality* dimension also was also narrowed down to include only *social functionality* because of the superior importance for patients and clinicians.

## Discussion

A dimensional structure of HrQoL *(emotional health, physical ailments, autonomy, social functionality, dignity*, and *resources*) for cancer patients in routine care was found based on four different sources (theoretical background, cancer patients, clinicians and experts).

One result of the first transcript’s analysis was a need for two additional inductive categories (*dignity* and *feeling of security in treatment*) to analyze all important information. Out of the nine deductive categories, four (*partnership/sexuality*, *spirituality/religiousness*, *health perception*, and *overall health*) were excluded after the qualitative content analysis was carried out. Two more categories, one deductive (*mobility*) and one inductive (*feeling of security in treatment*), were excluded based on the expert consensus-based statements. Likewise, part of the expert discussions’ outcome was the addition of a final dimension (*resources*). This resulted in six dimensions of HrQoL for oncological patients. Of these six dimensions, four (*emotional health*, *physical ailments*, *autonomy*, and *social functionality*) have been commonly used in HrQoL measurements for (cancer) patients (Ware and Sherbourne, 1992; Aaronson et al., 1993; Cella et al., 1993, 2010; Kohlmann et al., 1997; Gunzelmann et al., 2002; Mehnert et al., 2006; Herdman et al., 2011).

One essential outcome of this study was the importance of the dimension *dignity*. Both patients and clinicians voiced the importance of dignity, particularly during cancer treatment. Especially in a hospital setting it is important to encourage communication about patients’ sense of dignity as it affects patients’ HrQoL as well as the clinician-patient relationship. This assessment is supported by several research findings (Chochinov et al., 2011; Samuel et al., 2019; Xiao et al., 2019). Samuel et al. (2019) found that patients who received great respect/dignity by their physicians were more likely to state better HrQoL than patients who received less than optimal respect/dignity. Established HrQoL questionnaires do not assess dignity directly although a questionnaire for measuring dignity-related distress exists (Sautier et al., 2014).

Regarding the dimension *social functionality*, the influence of the relationship with a partner was rated less significant than social relations in general and the ability to connect with friends and family.

“*Family plays a special role in terms of quality of life.*” REC037, *social functionality*, 05:21:00.

Next to partnership and sexuality, other areas of functionality were not considered as relevant in this study. This differs from expectations found in outcomes of other HrQoL measurement studies (Ware and Sherbourne, 1992; Aaronson et al., 1993; Cella et al., 1993). In the EORTC QLQ-C30 (Aaronson et al., 1993) for example, different aspects of functionality, e.g., physical, role, cognitive, and emotional functionality, play an important role. An explanation for the outcome of this study could be that patients were exclusively questioned in a hospital setting. Over half of the participants (60.7%) were inpatients at the time of the survey. During a hospital stay the focus and needs of the patients shift, so that for example work-related functionality in this context loses importance. The results of this study are a suitable example for the importance of the setting in which a questionnaire is developed. The difference to other clusters of HrQoL dimensions becomes apparent when regarding the issues of pain and mobility which are dimensions often used to measure HrQoL in (cancer) patients (Ware and Sherbourne (1992); Aaronson et al., 1993; Kohlmann et al., 1997; Gunzelmann et al., 2002; Cella et al., 2010; Herdman et al., 2011). One part of the certification as an oncological center in Germany is the daily measurement and documentation of the patient’s experience of pain. Hence, to avoid redundancies, no dimension nor item on pain is necessary in a dimensional system for an oncological certified medical center.

As expected, mobility was an important aspect for the study’s participants even though it was only mentioned in relation to the subjective higher goal of autonomy. So that *mobility* must therefore be seen as subordinate to *autonomy*.

“*Self-care/autonomy often goes hand in hand with mobility. Feeling of freedom is also important here, not being restricted in one*’*s actions*.” REC006, *autonomy*, 15:03:00.

These two aspects (autonomy and mobility) especially reflect on the contrast between a solely outpatient or research setting and a hospital setting.

An unexpected result of our study was the addition of the dimension *resources*. Even though resources/coping is important for disease management (Doege et al., 2019; He et al., 2019; Lai et al., 2020), so far it is seldom connected to HrQoL measurements (Ware and Sherbourne, 1992; Aaronson et al., 1993; Cella et al., 1993, 2010; Herdman et al., 2011). Doege et al. (2019) examined a wide range of resources (e.g., family and financial security) in long-term breast, colorectal and prostate cancer patients and found that a greater number of highly differentiated resources led to a higher HrQoL. The general inquiry into a patient’s resources and ability to cope, is one of two aspects the new resource dimension is intended to address. The other reason for adding this dimension is of a practical nature as it assists clinicians in determining a patient’s need for further support by for instance consulting a psycho-oncologist.

### Study Limitations and Strengths

Limitations of this study are the selective and partially low participation in the conducted focus groups. No focus groups with attendings could be conducted and focus group sizes were in part small (two to three participants). Both limitations were the result of time constraints among clinicians. Changes in study design were executed and face to face interviews with attendings were conducted to adapt to their needs. Another limitation is that not all text material was double coded. The resources were too limited to ensure double coding of all qualitative data.

The comprehensive study design in contrast is a strength of this study as it includes experiences from cancer patients, clinicians, and various experts. Furthermore, in- and outpatients with heterogenic cancer diagnoses were interviewed. Instead of asking patients a series of questions one clear main question was asked with the option of more detailed subsequent inquiry if necessary. This way free association on the patient’s side was promoted and aspects of HrQoL that had a high importance for the respondents were included. Another strength of this study design is the combination of patient interviews and clinician focus groups. By conducting interviews it was possible to focus on the individual while during the focus groups the attention was concentrated on the discussion between the participants which generated additional information.

## Conclusion

An important finding of this study is that the issues affecting cancer patients depend on and change within the setting they find themselves in. An implication for practice could be to assess whether accessible questionnaires are suitable for monitoring PROs. The implementation of a well evaluated measurement for HrQoL can be obstructed if the tested environment is mismatched. Additionally, HrQoL dimensions of emotional health, physical ailment, social functionality, autonomy, dignity, and resources seem to be important in a hospital setting for patients and clinicians alike and should be considered during treatment. Beyond a mere query about a patient’s condition, clinicians get to know a patient’s need for support. It will also be possible to derive actions from the outcomes of a newly developed questionnaire for physicians, nurses, social workers or psycho-oncologists.

A dimensional structure of HrQoL in cancer patients in routine oncological care was found, which differs from dimensional structures of existing HrQoL measurements for (cancer) patients. Further research is needed to test and evaluate the presented structure in a large sample of cancer patients to further assess its psychometrical quality criteria. On the grounds of this study, items will be phrased and a short questionnaire for HrQoL in cancer patients suitable for the use in routine care will be constructed and psychometrically evaluated.

## Data Availability Statement

The datasets generated and analyzed during the current study are not readily available due to interviewees not giving their consent to publication. Requests to access the datasets should be directed to TS, t.schrage@uke.de.

## Ethics Statement

The studies involving human participants were reviewed and approved by the Ethics Committee of the Medical Association Hamburg (no. PV5636). The patients/participants provided their written informed consent to participate in this study.

## Author Contributions

CB and HS participated in the design and coordination of the study, as well as the editing of the manuscript. MG and TS collected all data and performed the analysis. TS drafted the manuscript. All authors read and approved the final manuscript.

## Conflict of Interest

The authors declare that the research was conducted in the absence of any commercial or financial relationships that could be construed as a potential conflict of interest.

## Publisher’s Note

All claims expressed in this article are solely those of the authors and do not necessarily represent those of their affiliated organizations, or those of the publisher, the editors and the reviewers. Any product that may be evaluated in this article, or claim that may be made by its manufacturer, is not guaranteed or endorsed by the publisher.

## Footnotes

1
https://www.maxqda.com/

## Supplementary Material

Supplementary Appendix A Semi structured interview guide.

Supplementary Appendix B Guide for conducting focus groups.

## Abbreviations

PROs: patient-reported outcomes
HrQoL: health-related quality of life
EORTC: European Organization for Research and Treatment of Cancer
FACT-G: Functional Assessment of Cancer Therapy –General
SF-36: Short Form-36 Health Survey
DT: Distress Thermometer
SRQR: Standards for Reporting Qualitative Research
UCCH: University Cancer Center Hamburg.
