# Supplementary material for: Identification and Determination of Dimensions of Health-Related Quality of Life for Cancer Patients in Routine Care – A Qualitative Study
Source: Front Psychol. 2022 Mar 9;13:824099. doi: 10.3389/fpsyg.2022.824099 (PMC8959933; doi:10.3389/fpsyg.2022.824099)
Supplement: Supplementary Appendix A — Semi structured interview guide. [file Table_1.docx]

**Supplementary Material A**

**Introduction Interview Patients**

Thank you for participating in our interview study. (explanation of study design, information about audio recording, data privacy, consent form). We perform interviews with you and other patients but also with clinicians. These interviews are part of a larger study. Our aim is to develop a questionnaire that measures Quality of Life for cancer patients. This questionnaire should be short and used regularly during treatment. The intention is that doctors, nurses, social services and psychooncologists are informed about more than just your physical ailments so that they can better support you.

**Interview guide**

| Leading question  (narrative prompt) | Check questions | Specific questions |  | Questions for continuity and navigation |
| --- | --- | --- | --- | --- |
| *part 1*  *introductory questions* |  | Have you ever participated in an interview? | |  |
| *part 2*  *Health-related Quality of Life*  “If you imagine that your current doctor asks you about your quality of life, physical and mental distress, what would be important for you in this matter? / What should not be omitted?“  „What would be important for you to ask in such an inquiry about quality of life?“ | What does quality of life mean to you?  What is particularly important to you?  What are issues that should be taken more into account by your practitioners (doctors, nurses, psychooncologists) or counsellors (social services)? | What are the burdens of your disease? What is a particular burden for you? What also helps you?  From your point of view, what would have to happen for doctors and nurses to receive information about your problems, fears and worries, beyond physical complaints? | | Could you elaborate that in more detail? |
| *part 3*  *Implementation*  “Just now it was more about what should be asked. Now I am still interested, when would be a suitable time to ask about your burdens? And how should the inquiry be conducted?“ | (Explain different possibilities of questionnaire survey) What would be the best/most suitable option for you? (paper-pencil, tablet, at the bedside)  Under which conditions would you be willing to complete a questionnaire?   - time, feedback of practitioner, length, presentation, fit, who should investigate | When would an inquiry not be helpful for you? (offering: length, layout, …)  What experience have you had so far with questionnaires? | |  |
| part 4  Additional inquiries |  | Did you come up with anything important during the interview that you would like to add? | | |
