# Supplementary material for: Identification and Determination of Dimensions of Health-Related Quality of Life for Cancer Patients in Routine Care – A Qualitative Study
Source: Front Psychol. 2022 Mar 9;13:824099. doi: 10.3389/fpsyg.2022.824099 (PMC8959933; doi:10.3389/fpsyg.2022.824099)
Supplement: Supplementary Appendix B — Guide for conducting focus groups. [file Table_2.docx]

**Supplementary Material B**

**Guide for focus groups**

Short introduction of moderators and the project PRO-ONKO-Routine, introduction of intention of this focus group, invitation to discuss freely, information about recording of the discussion, data privacy, consent form

**Opening Questions**

- Introduction round, facts, short answers (30sec), no power status highlighting

1. Please state your name and the ward or clinic you currently work at.

**Introductory Questions**

- Introducing topic, encourage conversation, open-ended questions, clues about participants views

1. What defines quality of life for you (in general and in oncological setting)?

**Transition Questions**

- Move conversation into key questions of study, go into more depth, participants become aware of others opinion on the topic, connection between participant and the topic

1. When thinking about your experience with oncological patients, what are important aspects of quality of life in cancer?

**Key Questions**

- Drive the study, 2-5 questions, 10-25 min each, after 1/3 - halfway through the focus group

1. Which aspects of quality of life do you consider to be particularly relevant to measure them with a short instrument?
2. What terms and conditions in the clinics and for practitioners are necessary so that the results of an instrument for quality of life can be used for patient treatment?
3. What are the obstacles to the use of an instrument for QoL in everyday clinical practice? (length of questionnaire, survey method, feedback of outcomes, ...)?

**Ending Questions**

- Closure, enable participants to reflect
- All things-considered question: determine final position on critical areas of concern, which aspects of discussion are most important/most in need of action 🡪 frequency in discussion does not reflect importance
- Summary question: after moderator has given a short summary (2-3 minutes) of the discussion, then participants are asked about adequacy
- Final question: ensure that critical aspects have not been overlooked, short overview of study, then “have we missed anything?”, if needed “how can we improve?”

1. When you think back to all the things we have talked about today, which aspects are particularly important to you?
2. SUMMARY (Co- Moderator)
3. Did xy correctly summarize, what we discussed today?
4. Is there something we missed or something we should have talked about?
5. Do you have any suggestions for future focus groups on what we should change or improve?
